# The Relation Between Attention and Tic Generation in Tourette Syndrome

**DOI:** 10.1037/neu0000161

**Published:** 2014-12-08

**Authors:** Erman Misirlisoy, Valerie Brandt, Christos Ganos, Jennifer Tübing, Alexander Münchau, Patrick Haggard

**Affiliations:** 1 Institute of Cognitive Neuroscience, University College London; 2 Department for Pediatric and Adult Movement Disorders and Neuropsychiatry, Institute of Neurogenetics, University of Lübeck and Department of Neurology, University Medical Center Hamburg-Eppendorf (UKE); 3 Institute of Cognitive Neuroscience, University College London and Department of Neurology, University Medical Center Hamburg-Eppendorf (UKE); 4 Department for Pediatric and Adult Movement Disorders and Neuropsychiatry, Institute of Neurogenetics, University of Lübeck; 5 Institute of Cognitive Neuroscience, University College London

**Keywords:** Tourette syndrome, tics, attention, inhibition, voluntary action

## Abstract

***Objective:*** Many neuropsychiatric disorders involve abnormal attentional processing. Systematic investigations of how attention may affect tic frequency in Tourette syndrome are lacking. ***Method:*** Patients performed rhythmic finger movements, approximately once every 2 s. Each movement triggered a unique visual color stimulus. Patients were asked to monitor and remember their finger actions, the external colors caused by their actions, or their tics. Sixteen adult Tourette syndrome patients performed each task twice: once while inhibiting tics, and once without inhibiting tics. ***Results:*** During the “freely tic” condition, patients had significantly fewer tics when attending to finger movements, or to the ensuing colors, compared with when attending to their tics. Attention to fingers produced the fewest tics overall. During tic suppression, tic frequency was reduced to an equal level in all conditions. ***Conclusions:*** Focusing attention away from tics significantly reduces tic frequency. This attentional process may operate by regulating motor noise.

Tourette syndrome (TS) is a neuropsychiatric disorder in which patients present multiple motor tics and at least one phonic tic for more than one year with onset before the age of 18 (*DSM–IV–TR*; American Psychiatric Association, 2000). Tics typically start around 6–7 years of age (Freeman et al., 2000; Robertson, 2000). Stress and anxiety are known to exacerbate tics (Conelea & Woods, 2008; Robertson, 2000), but the reasons for this interaction between state of arousal and tic generation remain unknown. Cognitive–behavioral theories postulate “vicious cycle” processes for anxiety disorders, in which excessive attention and sensitivity to anxiety-related body signals leads to increasing symptoms of anxiety (Clark, 1986; Taylor et al., 1992). We propose that a similar mechanism may underlie tic occurrence: excessive attention to tics, leads to a cycle of increasing tic frequency. This hypothesis has yet to be tested experimentally, to our knowledge. Here we report an experiment in which patients’ focus of attention was manipulated either toward their tics or to other stimuli while they performed a voluntary motor task. We investigated whether tic frequency varied with attentional focus, predicting that diverting attention away from tics would prevent enhancement of neuromotor noise and therefore reduce tics.

## Method and Materials

### Participants

Sixteen adult Tourette syndrome patients (mean age = 31 +/− 10.2 *SD*; 15 male) participated in the experiment with ethical approval. All procedures were in accordance with the Declaration of Helsinki. Patients were recruited in the Department of Pediatric and Adult Movement Disorders and Neuropsychiatry, Institute of Neurogenetics, University of Lübeck, Germany. All patients were diagnosed with Tourette syndrome according to *DSM–IV–TR* criteria (1). No patient fulfilled *DSM–IV* criteria for a diagnosis of obsessive–compulsive disorder (OCD).

TS symptom severity for the last week before testing was assessed by a clinician using the Yale Global Tic Severity Scale (YGTSS; Leckman et al., 1989). Premonitory urges were measured using the validated German version of the “Premonitory Urge for Tics Scale” (PUTS; Rössner et al., 2010; Woods et al., 2005), which assesses the quality, as well as the severity of bodily sensations preceding tics. Symptoms of attention-deficit hyperactivity disorder (ADHD) were rated on the German short version of the “Wender Utah Rating Scale” (WURS-K; Ward et al., 1993). Symptoms of obsessive–compulsive disorder (OCD) were rated on the “Yale-Brown Obsessive Compulsive Disorder Scale” (Y-BOCS; Goodman et al., 1989).

At the time of the study, all patients reported having motor tics and an additional eight reported having vocal tics. The mean YGTSS total tic severity was 16.8 +/− 7.7 *SD*, the mean YGTSS motor tic severity was 12.4 +/− 3.5 *SD*, and the mean YGTSS vocal tic severity was 4.4 +/− 5.5 *SD*. All patients reported premonitory urges. The mean PUTS score was 24 +/− 5.7 *SD*. Three patients were taking medication for their tics. Values of the Y-BOCS ranged from 0–11 (overall cut-off for OCD = 16), the mean total Y-BOCS score was 2.3 +/− 3.9 *SD*. Values of the WURS-K ranged from 0–44 with a mean of 16.8 +/− 12.1 *SD*. According to the WURS-K cut-off value of 30, three patients scored in the clinical range but only one of those patients fulfilled *DSM–IV* criteria for ADHD.

### Apparatus and Materials

The experiment was run on a computer. A video camera recorded the head and shoulders of each patient. The recordings were used for counting tics. Finger pressure sensors were attached to each of the four fingers of their dominant hand. These provided a digital output each time one of the four fingers was opposed to the thumb.

### Design and Procedure

**Figure 1 fig1:**
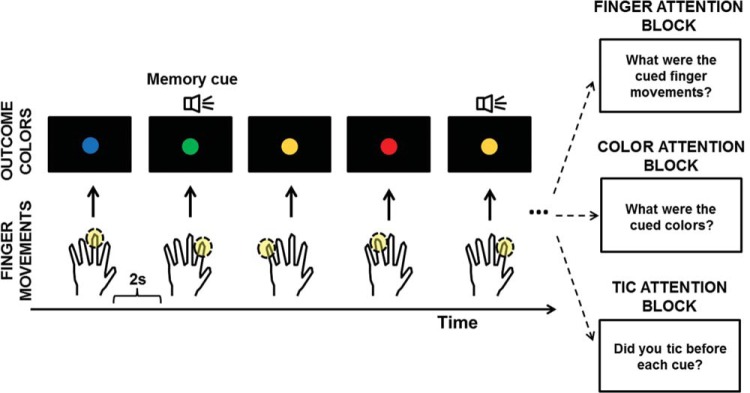
Key features of the experimental design. Patients opposed one finger of their choice, against the thumb, every 2 s. Each opposition produced a color on-screen. When random auditory “remember this” cues occurred, they had to remember a corresponding item. This was the finger moved, the color displayed, or the occurrence of a tic, according to condition.

The key features of the design are shown in Figure 1. Patients were asked to oppose a finger that they freely chose against the thumb of their dominant hand. They chose anew, and repeated the finger opposition approximately once every 2 s. They were asked to avoid using the same finger for consecutive actions, and to avoid adopting specific strategies or patterns of responses. Instead, patients were asked to decide spontaneously on a new finger for each action. Each trial lasted 1 min, giving approximately 30 actions per trial. An initial practice trial used an auditory metronome at a rate of 0.5 Hz to allow patients to synchronize their pinching actions and become familiar with a 2-s rhythm. Practice data was not recorded or analyzed.[Fig-anchor fig1]

Each time patients performed an action, a large colored circle immediately appeared in the center of the laptop screen for 750 ms. There were four possible colors (blue, green, red, and orange). The sequence of colors was random, except that a color could not be repeated twice in a row. The finger used for each action, and the color seen on-screen were completely independent.

In each 1-min trial, a 100 ms auditory tone was presented 250 ms after the onset of some colors. This tone instructed participants to remember a prespecified item. Each trial contained three, four, or five such tones, randomly and equiprobably. The timing of tones within trials was random, so the to-be-remembered items occurred at random times within each trial. The to-be-remembered items depended on the condition. Patients were asked to focus on, and remember, either the finger they used when they heard the beep (*finger condition*), the color they saw when they heard the beep (*color condition*), or whether they had produced a tic between the previous color and the current tone during the approximate 2-s interval (*tic condition*). We emphasized to patients in our instructions that they should focus their attention entirely on the items that they needed to remember in the current condition, and that they should ignore other items. Focusing on items irrelevant to the current task would have been disadvantageous, and patients were specifically told that they would perform best by maintaining attention to *only* fingers, *only* colors, or *only* tics, depending on the condition.

In summary, a voluntary motor finger-opposition task was performed in all conditions. Conditions only differed in requiring patients to focus their attention on perception of their fingers, external colors, or their own tics. A memory task was used to verify that participants complied with the task instructions. Attention conditions were blocked so patients always knew which events to attend to on each trial. They were asked to remember the correct item for every “remember this” tone, in each trial, in the correct order.

At the end of the trial, patients were first asked how many tone memory cues had occurred. The next question varied according to condition. In the *finger attention* condition, they were asked to report the finger used when each beep occurred during the trial. They responded motorically, by opposing the appropriate fingers against the thumb in the correct order, to reconstruct the memory sequence. In the *color attention* condition, they were instead asked about the colors and entered their responses by typing “r” for red, “g” for green, “b” for blue, or “o” for orange on the computer keyboard, again in the order of their occurrence. In the *tic attention* condition, they typed a sequence of “j” for yes, and “n” for no, to indicate whether or not they produced a tic before each beep presented. After entering their responses, they continued with the next trial after pressing return on the keyboard.

In addition, we manipulated tic control by instruction. Patients performed each of the three conditions (a) while being instructed to voluntarily inhibit their tics to the best of their ability, and (b) without such instruction, so they could tic freely. This created a 3 × 2 repeated measures design, in which the first factor was the attentional manipulation and the second factor was instructed tic control. Nine 1-min trials were performed in each condition of this design, giving a total of 54 trials across the experiment. The main outcome measure was tic frequency in each condition. Our main research questions were: whether attentional focus influenced tic frequency, and whether this mechanism was related to voluntary tic inhibition, or was rather independent of it.

### Tic Counting

Before the main tasks in the experiment, each patient was video recorded at rest for 1 min, without instruction regarding tics. In addition, a further 1-min recording was made after giving the instruction to voluntarily inhibit tics. These baseline conditions were always performed in this order. This was to avoid any potential ‘rebound’ effect of increased tics following tic inhibition which patients often report (Verdellen et al., 2007). The number of tics in these trials was used as a baseline measure of tics, to compare with tic frequency during trials in the experiment.

Two experimenters independently counted tics in all videos for all conditions. The mean of their independent counts was used in all analyses. These experimenters were blind to all experimental conditions during tic counting but not to experimental hypotheses or design. A third rater naïve to all aspects of the experimental design including hypotheses and conditions independently counted a subset of tic videos (the first five trials from one condition drawn at random from each patient’s data). Their count was used to test interrater reliability with the counts used for analysis.

## Results

### Behavioral Data

Patients generally had no difficulties in maintaining the 2-s rhythm with finger pinching actions. The mean interval between their finger movements was 1.79 s (average standard deviation over movements of 0.30 s). Patients also avoided using the same finger consecutively in 96.21% of their actions, as instructed.

Memory performance was high across all finger and color conditions (see Table 1). A 2 × 2 repeated measures ANOVA on memory performance showed no main effect of attentional focus, *F*(1, 15) = 2.9, *p* = .11, no main effect of tic inhibition, *F*(1, 15) = 0.02, *p* = .88, and no significant interaction, *F*(1, 15) = 0.56, *p* = .46. This suggests that finger and color tasks did not differ significantly in difficulty. Measures of objective memory performance were not possible for the tic attention task for several reasons. First, patients’ perceptions of their own tics may be very different to observers’ perceptions (Müller-Vahl et al., 2014; Pappert et al., 2003). Second, patients may perceive tics in body regions not recorded by the video camera. Third, any false positives and misses by the experimenter during tic counting would greatly influence measures of memory performance for tics. For these reasons, a reliable estimate of memory for tics based on matching tic rater and patient reports is impossible in the context of the current experiment.[Table-anchor tbl1]

### Tic Frequency

A strongly significant correlation between independent raters’ tic counts was found (*r* = .94, *p* < .001, see supp. Figure 1), suggesting that they produced highly similar data following tic counting.

**Figure 2 fig2:**
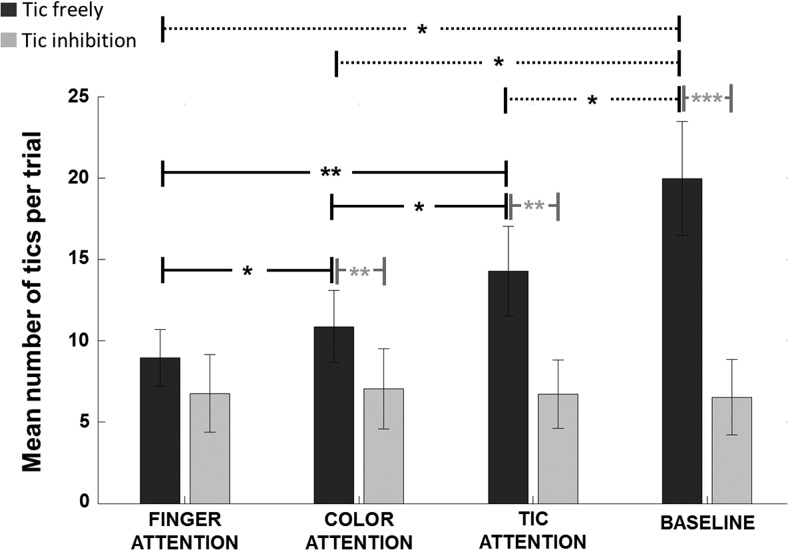
Mean number of tics per trial for each task condition and baseline. Black connecting lines indicate significant differences between freely tic conditions, and gray connecting lines indicate significant tic reductions when inhibiting tics within an attention condition (* *p* < .05. ** *p* < .01. *** *p* < .001). The freely tic baseline had significantly greater tics than all within-task freely tic conditions (dashed lines, *p* < .05, Bonferroni corrected). There were no differences between baseline tic inhibition and within-task tic inhibition conditions. Note that no difference was found between freely tic and tic inhibition states in the finger attention condition.

The mean number of tics per trial in each task condition is shown in Figure 2. A 2 × 3 repeated measures ANOVA showed a significant main effect of attention, *F*(2, 30) = 6.54, *p* = .004, and a significant main effect of tic inhibition, *F*(1, 15) = 9.27, *p* = .008. A Mauchly’s test of sphericity indicated a significant violation for the Attention × Inhibition interaction (*p* < .05). After applying a Greenhouse-Geisser correction, a significant interaction was found, *F*(1.41, 21.09) = 5.31, *p* = .02. We explored this interaction with simple effects *t* tests. These results are shown in Figure 2.[Fig-anchor fig2]

When allowed to tic freely, patients produced the highest number of tics in the tic attention condition during task performance. Tics were significantly reduced when patients focused their attention on colors compared to tics, *t*(15) = −2.16, *p* = .047 (effect size: Cohen’s *d* = 0.34). Tics were reduced even further when focusing attention on fingers relative to colors, *t*(15) = −2.15, *p* = .048 (Cohen’s *d* = 0.24). Interestingly, tic frequency was at an identical level for all attention conditions when patients were asked to inhibit their tics. When comparing with the passive baseline in which patients performed no task, it can be seen that tic inhibition during tasks reduced tics to an “inhibition baseline” level. Individual *t* tests between the inhibition baseline and each of the three tic inhibition task conditions, showed no significant differences even without correcting for multiple comparisons (*p* > .05). In contrast, Bonferroni corrected *t* tests showed that the baseline tic frequency during free tics was significantly higher than all three of the free tic task conditions (*p* < .05). This suggests that the attentional demands of any general task performance (e.g., maintaining a steady rhythm in actions; remembering items) reduced tics to some extent. The important point is that the level of tic reduction was significantly affected by the focus of attention.

## Discussion

Participants were asked to attend to, and count, specific events. The nature of the events attended strongly influenced tic frequency. The results highlight the important role that attention plays in the presentation of tics. Simply engaging attention in a task reduced the frequency of tics from a baseline level with no task. This is consistent with a general distracting effect of any cognitive task. However, the specific content of attention strongly influenced tic frequency. Tics were most frequent when attending to tics, least frequent when attending to voluntary finger movements, and showed an intermediate level when attending to an external stimulus caused by the participant’s voluntary finger movements. These findings highlight that Tourette syndrome should not be viewed as a unitary motor disorder (Cavanna et al., 2009). Indeed, cognitive impairments are frequently reported in TS, particularly in relation to executive function and sustained attention (Eddy & Cavanna, 2014; Eddy, Rickards, & Cavanna, 2012; Eddy, Rizzo, & Cavanna, 2009; Jeter et al., 2014). Our findings agree with this view, by demonstrating the importance of attentional allocation in TS symptoms.

### Attention and Motor Noise

The term “neural noise” generally refers to spontaneous changes in brain activity, which are not related to any particular experimental task or identifiable stimulus (Fox & Raichle, 2007). Interestingly, some degree of neural “noise” could have an important functional role, so such neural activity should not simply be dismissed as irrelevant or redundant (Fox & Raichle, 2007; Garrett, Kovacevic, McIntosh, & Grady, 2010; Garrett, Kovacevic, McIntosh, & Grady, 2011; Garrett et al., 2013; McDonnell & Ward, 2011). Neural noise is naturally present in the motor system and can affect motor planning and movement (Hamilton et al., 2004; Harris & Wolpert, 1998). However, normal context-embedded actions depend on voluntary planning and intention, rather than motor noise. Attention enhances neural responses in brain areas that code for attended objects (Kastner et al., 1998; Moran & Desimone, 1985; Murray & Wojciulik, 2004; Saalmann et al., 2007; Somers et al., 1999; Treue & Maunsell, 1996). In principle, such attentional facilitation could also enhance tic-related neural activity. We propose that motor noise in TS patients is greater than in healthy people, and that such noise processes contribute to tic generation. Excessive attention to these involuntary motor processes could increase involuntary motor activity, and therefore tic frequency.

Tics are very common in young children with prevalence estimates up to 18% in the early school years (Ludolph et al., 2012; Robertson, 2000). Tics (i.e., superfluous and repetitive context-independent actions) might be a reflection of increased motor noise and perhaps the product of an immature motor system going through a “tuning” process. They are often not noticed and not troublesome, and typically subside within a few months. However, in some children and adolescents, tics persist and may cause distress. When motor and phonic tics are present for more than a year, this is referred to as Tourette syndrome. Why tics persist in some patients is unclear. Some possible factors have been proposed, for example streptococcal infection (Allen et al., 1995; Mell et al., 2005; Toufexis et al., 2014), but none have been conclusive or widely accepted. The role of attention in tic persistence has not been studied systematically. The results of the present study show that attention enhances tic generation. It may further be conceivable that increased attention to tics by relatives, teachers and peers, could also promote tic persistence, by directly influencing how much the patient pays attention to his or her tics.

The results of the present study show that distraction of attention away from tics reduces tic frequency. Tic reductions were found in all attention conditions relative to baseline. Task demands such as retaining information in memory or maintaining a steady action rhythm, are sufficient to divert attention from tics to some extent. These were common to all tasks, including the tic attention task. However, the extent of distraction could be improved by manipulating the specific object of attention. Selective attention to the color of the stimuli presented on the screen meant that attention was focused on external stimuli, and allocated away from tics. We propose that this prevented attentional facilitation of tic-related neural signals (Woodman & Luck, 2003). The distraction provided by attending specifically to finger actions during the task appeared to exhibit even greater tic reduction benefits. This may be because attention is focused on voluntary as opposed to involuntary actions. Some have argued that the boundary between voluntary and involuntary actions becomes blurred in TS (Cavanna & Nani, 2013). Our data suggest that attention to voluntary action generation may lead to a stronger separation of the two systems. Attention to voluntary actions may draw attentional resources away from involuntary movement. This focus on the voluntary stream of action may further inhibit any contribution to overt action from the involuntary stream.

Previous models have suggested multiple, dissociable cortical streams for action control. For example, internal self-generated and external stimulus-triggered actions are thought to involve different pathways (Jenkins et al., 2000). However, the pathway for tic generation remains controversial (Ganos et al., 2013). Our results argue against the view that tic generation originates in the voluntary “self-generated” motor pathway. In other words, the immediate urge to tic does not have a common origin with typical intention associated with voluntary actions. Had that been the case, attention to voluntary action and attention to tics should have comparable effects on tic frequency. In fact, a clear dissociation was found. Thus, our results support a dissociation, and even an inhibitory link between the voluntary and tic pathways, which can be modulated by attention. Recent neuroimaging findings propose a similar inhibitory interaction between tic inhibition and voluntary action pathways (Ganos et al., 2014; Thomalla et al., 2014). Voluntary movements are those which we associate with an immediate conscious intention to perform a movement, while involuntary movements are those which we perform automatically without any clear awareness or intention to move. We propose that tics in TS originate from the involuntary action stream, and that this system is “hyperactive,” in that it creates frequent and exaggerated context-irrelevant actions.

An alternative interpretation states that there is a single system underlying both involuntary and voluntary actions. In TS, attention to tics prioritizes involuntary outputs and so tics dominate the available resources within the system and tic frequency increases. However, there is evidence for *multiple* routes to action. For example, voluntary and stimulus-driven actions respectively depend on medial frontal and lateral frontal inputs to primary motor cortex (Deiber et al., 1999). Tics might originate in just one such independent stream. We found that attention to voluntary actions does not facilitate tics and in fact reduces them, while attention to tics increases their occurrence. This pattern of results is consistent with the view that tics do not arise from the same pathway as voluntary actions.

### Tic Inhibition

The instruction to voluntarily inhibit tics was generally effective consistent with previous studies (Ganos et al., 2012; Peterson et al., 1998; Serrien, Orth, Evans, Lees, & Brown, 2004). This instruction brought tics down to the same low baseline level in every attentional condition, despite large differences between attentional conditions in the number of tics without inhibition. This suggests that tic inhibition is applied at the final motor output stage of tic generation. Any prior influences on the tic generation process might be simply cancelled out by a “cut-off” mechanism which acts to block the output stream and reduce or prevent overt tic expression. Tic inhibition is thought to rely on a network of basal ganglia, thalamus, and prefrontal areas (Peterson et al., 1998; Serrien et al., 2004). Interestingly, inhibitory activity increases as tic onset time approaches (Serrien et al., 2004), suggesting that inhibition is continuously regulated to adaptively control tic frequency. Although tic suppression is successful in reducing tics, it may not remove the urge to tic. Overall, there is no correlation between trait level of urge to tic as measured by PUTS and tic inhibition (Ganos et al., 2012; Müller-Vahl et al., 2014). However, tic inhibition in some patients may in fact produce a continuing intensification of the immediate urge to tic (Himle et al., 2007). The mechanism of tic reduction by attentional focus is likely to be quite different to tic inhibition. We have suggested that attention may operate by modulating noise at the tic generation stage. In contrast, we suggest that inhibition is applied at a later stage, gating the output of the generator. Therefore, reductions in tic frequency through attentional manipulation seem to occur without the need for effortful inhibition of tics. Note that the number of tics during attention to voluntary finger movements in the current task was reduced to the same extent as active tic inhibition (see Figure 2). We show that patients may be able to obtain tic reduction benefits as strong as those with tic inhibition, without the need for continuous internal monitoring and active tic prevention.

### Attention in Other Disorders

Several other psychiatric disorders involve an attentional component. Anxiety and panic disorders involve excessive attention to internal body signals and misinterpretation of normal bodily processes and sensations (Clark, 1986; Taylor et al., 1992). Psychogenic disorders have also been characterized by maladaptive attentional processes (Edwards et al., 2012; Edwards & Bhatia, 2012). For example, attention to abnormal prior beliefs about bodily sensations and movements excessively weights their contribution to perception and action, creating the functional symptoms of movement disorders (Edwards et al., 2012). Psychogenic movement disorders are of course very different to TS, both in etiology and in phenomenology. However, our data suggest that maladaptive attention in TS may exacerbate tic symptoms. For example, it might augment random fluctuations in motor noise, generating urges to tic that might not occur in the absence of attention.

### Implications for Behavioral Treatment

The data we present here has several implications for behavioral treatments of TS. One important current behavioral treatment is habit reversal therapy (HRT; Bate et al., 2011; Deckersbach et al., 2006; Himle et al., 2006; Wilhelm et al., 2003). This therapy aims to reduce the association between urge and tic by encouraging patients to become more aware of their tics as they occur, and teaching them to produce a movement which is incompatible with the urge to tic. It appears to work through improving the ability to control and inhibit tics (Bate et al., 2011). Although HRT and other existing behavioral interventions show some success, response rates are often suboptimal in both adults (Wilhelm et al., 2012) and children (Piacentini et al., 2010). For example, 10 months after HRT, patient tic severity does not differ significantly from control patients who were given supportive psychotherapy treatments (Wilhelm et al., 2012). In contrast to HRT, our results might suggest a treatment based on attentional distraction. This putative therapy would minimize attention to tics, with the aim of reducing the urge to tic in the first place, rather than improving the control of overt tics. Instead of teaching patients to become highly aware of the warning signs of a tic, as HRT does, patients would be encouraged to focus their attention on external events and fully voluntary actions and intentions. By employing these distraction techniques under conditions in which they are most likely to tic, such as anxious and stressful social situations, patients may be able to reach a level of tic frequency similar to effortful tic inhibition without having to actively control and monitor involuntary urges. This putative therapy remains speculative, but it is clearly suggested by the evidence presented here.

### Limitations

We lacked any subjective self-report measures of online urge during the task from patients. We have suggested that attentional distraction reduces urges to tic, but we have no direct measure to show this. However, the way in which tic frequency changed across attentional conditions was very different from the way it changed with tic inhibition. Tic inhibition consistently reduced tics to the same level, whereas attentional distraction had a more graded effect depending on the quality of the distraction. We believe there are two possible ways to reduce tics: either actively inhibiting them, or having a reduced urge to tic in the first place. While tic inhibition involves the first mechanism, attentional distraction should involve the second.

A second potential limitation is the possible confound of task difficulty. We could not directly measure task difficulty in the tic attention condition due to the inability to accurately categorize a patient’s tic judgments as correct or incorrect (see Results). However, we can certainly rule out task difficulty as an explanation of the difference in tic frequency between finger and color conditions. Objective measures of task performance did not differ between these conditions. Therefore, attention alone can affect tic production, irrespective of the difficulty of the attended task. Although the tic attention task may be easier overall than finger and color tasks, task difficulty alone cannot explain our data on changing tic frequency with attention.

## Conclusions

In conclusion, we provide evidence that tic generation is strongly influenced by attention. When patients attend directly to tics, they exhibit a higher number of tics than when attending to other events or objects. An active desire or instruction to stop tics further facilitates attention to tics, potentially creating a cycle of increasing symptoms. This process may also explain the reported increase in tics during stress outside the laboratory (Conelea & Woods, 2008), because tics may become the focus of attention in such situations, particularly when there is some social relevance. We propose that directing attention away from tics may improve the signal-to-noise ratio in the motor system. This ultimately means that patients tic less than they normally would, without the need to use effortful tic suppression.

## Supplementary Material

10.1037/neu0000161.supp

## Figures and Tables

**Table 1 tbl1:** Percentage of Correctly Recalled Items in the Finger and Color Tasks During Freely Tic and Tic Inhibition Conditions

	Finger	Color
Freely tic	79%	89%
Tic inhibition	82%	86%
