# Supplementary material for: The Relation Between Attention and Tic Generation in Tourette Syndrome
Source: Neuropsychology. 2014 Dec 8;29(4):658–65. doi: 10.1037/neu0000161 (PMC4484548; doi:10.1037/neu0000161)
Supplement: Supplementary file 1 [file z6o006142747so1.docx]

**Supplemental Materials**

**The Relation Between Attention and Tic Generation in Tourette Syndrome**

**by E. Misirlisoy et al., 2014, *Neuropsychology***

**http://dx.doi.org/10.1037/neu0000161**


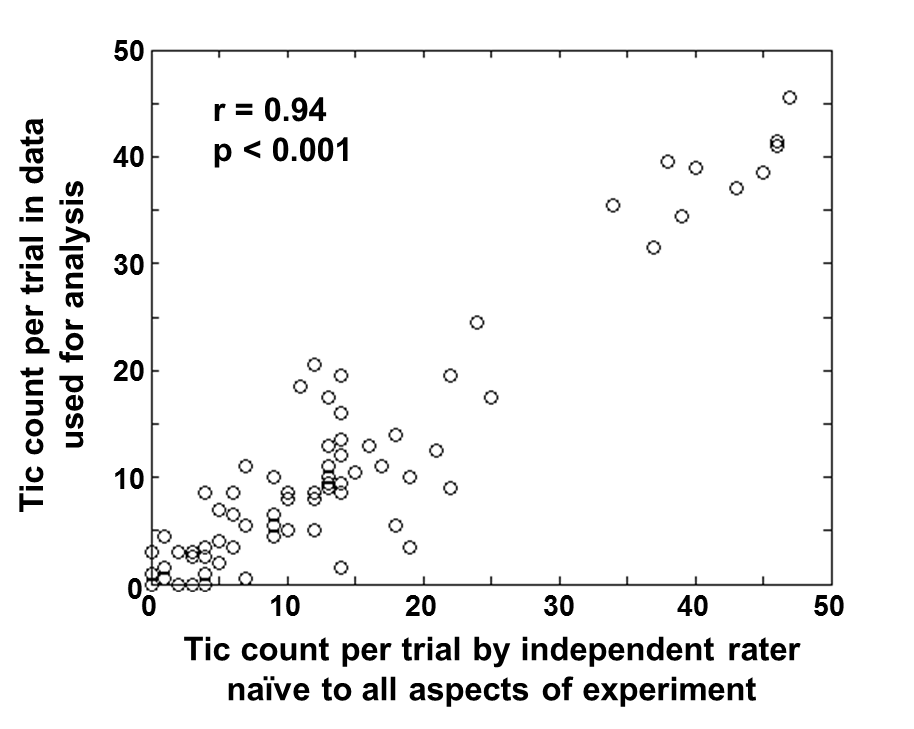


***Figure S1*.** Inter-rater reliability between rater counts used for analysis and independent rater naïve to all aspects of the experiment. Each datapoint is the tic count for a single trial across a subset of 80 trials. Ratings on the y-axis show the mean of independent counts performed by authors EM and VB. Ratings on the x-axis represent counts performed by an independent rater not involved in the experiment.
